# The Years at the Seamen's Hospital

**Published:** 1920-05-08

**Authors:** P. J. Michelli


### THE YEARS AT THE SEAMEN'S HOSPITAL.

**By P. J. Michelli, C.M.G.**

It was in February, 1878, that I first met Sir Henry Burdett. He had then been secretary of the Seamen's Hospital Society for three years. I went with an introduction from Mr. Lovelace, then Director of Greenwich Hospital, who had heard that there was a subordinate post vacant in the Dreadnought Hospital, and I meant to be a candidate for that. I soon found that I was in the presence of an indomitable personage. His piercing blue eyes and keen searching manner were not calculated to put a novice at his ease. He soon turned me inside out, disclosed my absolute ignorance of affairs, and I left, somewhat bewildered, but convinced that I had met a man with whom I could work and who would require of me the uttermost. That in a word sums up his chief characteristics. Work he did, work of the most strenuous, but he required a like product in those with whom he was associated. Unsparing of himself, he was unsparing of others, and the task was never done until his dying day. He had found the old Dreadnought wending its quiet way, keeping up an old and quiet tradition, but without progress. He had done much and was doing more. The income rose from the first year he joined. Improvements had already been made, improvements in the nursing, improvements in the management, and the spirit of advance was infused into the old hospital.

No detail was too small for his keen perception. By the year 1881, when he left, the income had nearly doubled and the Dreadnought could claim to be in the van of the London hospitals and a model to the great majority. He grasped the fact that money is seldom given solely because the plea is good, and was the first to realise that the amount contributed is more in proportion to the importance of the applicant than to the merits of the cause. Never was a man more able to spot the giver and to get at the giver. He knew the value of a great name, and was the first to get it put into practice. He induced the Duke of Northumberland to issue an appeal in his own name from his private address in Grosvenor Place, the contributions being sent direct to His Grace and the receipts being sent under the ducal signature; this brought a very substantial sum. He worked the Shipowners and every shipping interest, and so laid the foundation of that admirable income that the hospital now enjoys. With all these activities he yet found time for other spheres of labour. He was a medical student at Guy's during the greater part of the time he was secretary of the Hospital. He worked on schemes of statistics for comparing the expenditure of one hospital with another. He was ever writing contributions for the Press, and, what is more, they were published.

Then he left in 1881 for the Stock Exchange, but that was no check to his work for the hospitals. A year after, I was appointed secretary to St. Mary's, in close proximity to his home, and there we worked together harder than ever. Night after night, till one or two in the morning, first on the formation of the Hospitals' Association and then on the "Hospitals and Charities Year-Book." He was instigator and founder of the former and editor of the latter. The Hospitals' Association prospered, as all did to which he put his hand, and it stands to-day with triumphs to its credit. To it are due the amendments to the Mortmain Acts. There the Midwives Bill, the Uniform System of Accounts, which he originated, had their inception, and so on until at last the labourer's task is o'er.

It is not, perhaps, too much to say that the present highly organised voluntary system is in a large measure due to Sir Henry, and had he lived it is more than likely that he would have righted its financial straits. To him the hospitals in general, and the writer in particular, owe much.

A memorandum such as this should not close without some mention of the gracious lady whom he married at Greenwich in 1875—gentle, witty, courteous, unselfish, and a hostess past compare. Her *rôle* was not always the easiest—how could it be beside an avalanche of persistent toil?—but she had her consolations along with her trials, consolations that come to those who are able to accomplish much for others. Who can measure what he owed to her?

